# Artifacts in time-resolved Kelvin probe force microscopy

**DOI:** 10.3762/bjnano.9.119

**Published:** 2018-04-24

**Authors:** Sascha Sadewasser, Nicoleta Nicoara, Santiago D Solares

**Affiliations:** 1 International Iberian Nanotechnology Laboratory, Av. Mestre José Veiga s/n, 4715-330 Braga, Portugal; 2 The George Washington University, Department of Mechanical and Aerospace Engineering, 800 22nd St. NW, Ste. 3000, Washington, DC 20052, USA

**Keywords:** Kelvin probe force microscopy, time-resolved

## Abstract

Kelvin probe force microscopy (KPFM) has been used for the characterization of metals, insulators, and semiconducting materials on the nanometer scale. Especially in semiconductors, the charge dynamics are of high interest. Recently, several techniques for time-resolved measurements with time resolution down to picoseconds have been developed, many times using a modulated excitation signal, e.g., light modulation or bias modulation that induces changes in the charge carrier distribution. For fast modulation frequencies, the KPFM controller measures an average surface potential, which contains information about the involved charge carrier dynamics. Here, we show that such measurements are prone to artifacts due to frequency mixing, by performing numerical dynamics simulations of the cantilever oscillation in KPFM subjected to a bias-modulated signal. For square bias pulses, the resulting time-dependent electrostatic forces are very complex and result in intricate mixing of frequencies that may, in some cases, have a component at the detection frequency, leading to falsified KPFM measurements. Additionally, we performed fast Fourier transform (FFT) analyses that match the results of the numerical dynamics simulations. Small differences are observed that can be attributed to transients and higher-order Fourier components, as a consequence of the intricate nature of the cantilever driving forces. These results are corroborated by experimental measurements on a model system. In the experimental case, additional artifacts are observed due to constructive or destructive interference of the bias modulation with the cantilever oscillation. Also, in the case of light modulation, we demonstrate artifacts due to unwanted illumination of the photodetector of the beam deflection detection system. Finally, guidelines for avoiding such artifacts are given.

## Introduction

Kelvin probe force microscopy (KPFM) [1] has been widely used for the characterization of metals, insulators, and semiconducting materials on the nanometer scale [2]. The imaging mechanism relies on the compensation of electrostatic forces by application of a bias voltage that corresponds to the local contact potential difference (CPD), the relative difference between the work function of the tip and that of the sample area below the tip. In most applications, spatial variations of the CPD are imaged in a static fashion, where variations in the CPD images can have different origins. (i) Variations in the local surface structure, chemistry, or material can affect the CPD by means of a change in the surface dipole, the electron affinity, or the work function [3–5]. (ii) The CPD can reflect spatial variations in the charge density [6–8], individual localized charges [9], or even partial charge densities within a single molecule [10–11]. Finally, (iii) doping type and charge-carrier concentration in semiconductors will control the position of the Fermi level, affecting the work function, which is defined as the energy difference between the local vacuum level and the Fermi level [12]. Usually, KPFM is used as a slow technique aiming at imaging local variations in the CPD. The KPFM feedback circuit applies a dc voltage to the tip (or the sample) to compensate the electrostatic forces between tip and sample, where the time constant of the KPFM controller is typically in the range of milliseconds. However, especially in semiconductors, and in view of points (ii) and (iii), the charge dynamics are of high interest in materials and device characterization, and knowledge of the nanoscale charge-carrier dynamics can provide valuable insight into device functionality and limitations in device performance.

As a consequence, recently, several techniques for time-resolved measurements with time resolution down to picoseconds have been developed. In the simplest approach, time-dependent changes in the CPD are observed in real time in a point measurement following an excitation pulse. Sadewasser et al. [13] studied light-induced changes in a CuGaSe_2_ semiconductor used in photovoltaic applications. The authors measured the surface photovoltage (SPV) – the difference between the CPD under illumination and in the dark – after switching on and off a laser light source. The CPD change resulting from the separation of the excited charge carriers was monitored in real time over the course of several minutes. Similar experiments were also performed using electrostatic force microscopy (EFM) on organic photovoltaic blends [14–16]. By applying a bias pulse to the atomic force microscopy (AFM) tip, Schirmeisen et al. studied the ion transport in solid electrolytes [17]. By applying bias pulses across organic field-effect transistors (OFETs) electronic transport in organic materials was studied [18–21]. The time resolution in these approaches is limited by the KPFM controller (to typically the millisecond range) or the response time of the cantilever to changes in the sample’s CPD (on the order of 100 μs [14]).

A better time resolution was achieved by using a light-intensity modulated (IM) KPFM measurement, presented by Takihara et al. [22]. Charge carriers are excited during the illuminated fraction of the modulation, and decay during the subsequent dark fraction of the modulation. If the modulation frequency is faster than the response time of the KPFM controller, only an averaged CPD will be measured, for which the value will depend on how fast the changes in charge carrier separation follow the light modulation. Thus, the average CPD carries information about the charge carrier dynamics. This technique was subsequently used by various groups for the characterization of organic devices [23–26] and, by using bias modulation (BM) KPFM, also for the measurement of the minority carrier lifetime in epitaxial Si solar cell materials [27]. In a variation of the bias or light modulation approach, a bias-based pump–probe approach (pp-KPFM) was used to measure the charge-carrier dynamics with a time resolution of 2 μs in pentacene-based OFETs [28–29]. Similarly, light-based pp-KPFM was used to measure a charge carrier lifetime in low-temperature grown GaAs of ≈1 ps, currently the best time-resolution that has been demonstrated experimentally for KPFM [30].

In these fast KPFM approaches relying on the application of a modulated excitation signal (light, bias, or any signal that results in a respective CPD change), there is a possibility that for specific frequencies the excitation signal interferes in an unwanted way with the cantilever oscillation or the ac-voltage that is usually applied for the detection of the CPD. Such interference can be expected to affect the correct measurement of the CPD, leading to an artifact that could be misinterpreted as a sample property. To address this important issue, we have performed numerical dynamics simulations of the cantilever oscillation in KPFM subjected to a bias-modulated signal. We consider square bias pulses, as well as exponentially raising and falling pulses. For square pulses, the resulting time-dependent electrostatic forces are very complex and result in intricate mixing of frequencies that may in some cases have a component at the detection frequency (cantilever resonance frequency). When this happens, the measured CPD deviates from the expected value. Additionally, we performed fast Fourier transform (FFT) analyses that match the results of the numerical dynamics simulations. Small differences are observed in the measured CPD that can be attributed to transients and higher-order Fourier components, as a consequence of the intricate nature of the cantilever driving forces. Measurements on a model system (metal-coated tip and Au sample) confirm the simulation results. Furthermore, additional artifacts are observed due to an undesired influence on the *z* feedback controller and on the photodiode of the beam-deflection system in case of light modulation.

## Results and Discussion

### Simulations

Numerical simulations of the cantilever motion were performed using a C code. The cantilever tip dynamics are governed by the equation of motion:

[1]
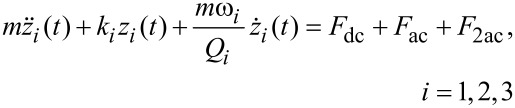


where *m* is the effective mass, *z* the vertical tip position above the sample surface (*z* = 0), *t* is the time, *k* the effective spring constant of the cantilever, ω the natural angular frequency of the cantilever, and *Q* the quality factor. The first three (*i* = 1,2,3) eigenmodes of the cantilever are used. Only the electrostatic forces relevant for electrostatic and Kelvin probe force microscopy are considered according to [2]:

[2]
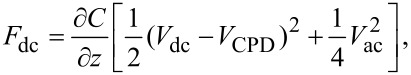


[3]
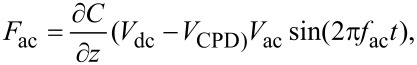


[4]
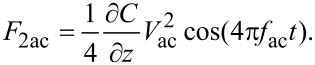


Here, *V*_dc_ is the applied dc voltage, *V*_ac_ the ac-detection voltage, *f*_ac_ the frequency of the ac-detection voltage and *V*_CPD_ the contact potential difference. In our numerical simulations, no *z* feedback is considered and the *z* position of the cantilever tip is only influenced by electrostatic forces. This was done in order to focus on the effect of the electrostatic forces.

To realize time-resolved KPFM a bias modulation in the form of a square voltage pulse train *V*_pulse_(*t*) is introduced as a perturbation to the tip–cantilever system by replacing *V*_dc_ in Equation 2 and Equation 3 with *V*_dc_(*t*) *= V*_dc_* + V*_pulse_(*t*), where:

[5]
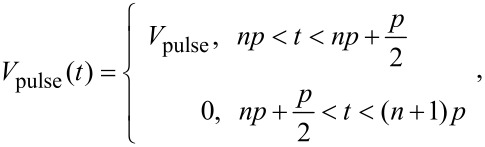


and *V*_pulse_ is the amplitude of the square voltage pulse, *p* is the period of the voltage modulation, and *n* is an integer (counter).

To explore any effects of the bias modulation in the CPD determined in a KPFM measurement, the CPD is considered constant, and for a given period, pulse amplitude, and dc bias voltage, the equation of motion is numerically solved and the amplitude of the tip oscillation at the ac-detection frequency (*f*_ac_) is extracted. This simulation is repeated for up to 30 different dc voltages in a range between −3 V < *V*_dc_ < +3 V. The expected v-shaped dependence according to Equation 3 is observed, where the minimum is extracted, which corresponds to the *V*_CPD_ that will be measured with the applied modulated square voltage pulses.

Typical values for the cantilever parameters were used, as given in Table 1. The simulations considered two typical cases: either the ac-detection voltage is applied on the (i) fundamental resonance frequency, or (ii) on the frequency of the second oscillation mode of the cantilever. We note that the quality factors used in the presented simulation results are rather small, even smaller than typical values obtained for AFM operation in ambient air. We used these small values since the calculation time depends on the quality factors and for larger values the duration of the simulations became rather long. Nevertheless, in several occasions, we tested the validity by using typical values as obtained in ultra-high vacuum conditions and always obtained the same results.

**Table 1:** Typical values used for the cantilever parameters in the simulations reported here.

Parameter	Value

force constant (N/m)	4.0
quality factor of fundamental eigenmode	750
quality factor of second eigenmode	450
quality factor of third eigenmode	150
capacitance gradient (N/V^2^)	1.0 × 10^−9^

The simulations of the tip–cantilever dynamics were performed for a variation of dc-bias voltages (to extract the measured *V*_CPD_) and for a variation of the period of the modulated bias voltage. While the simulations consider a direct modulation of the sample bias, this can correspond to experimental situations where the sample surface potential is modulated by an applied modulated bias, by modulated light pulses, or any other modulation that results in a modulation of the sample surface potential.

The numerical simulations only consider a slow Kelvin controller that cannot follow the applied bias modulation and therefore measures an average *V*_CPD_. With this in mind, the expected measured *V*_CPD_ would correspond to the sum of the predefined CPD and the time-averaged voltage from the modulated bias, which for a 50% duty cycle is half the amplitude *V*_pulse_: CPD + *V*_pulse_/2. Any deviation from this expected value can be assigned to an unwanted interference of the bias modulation with the oscillating tip–cantilever system.

**Figure 1:**
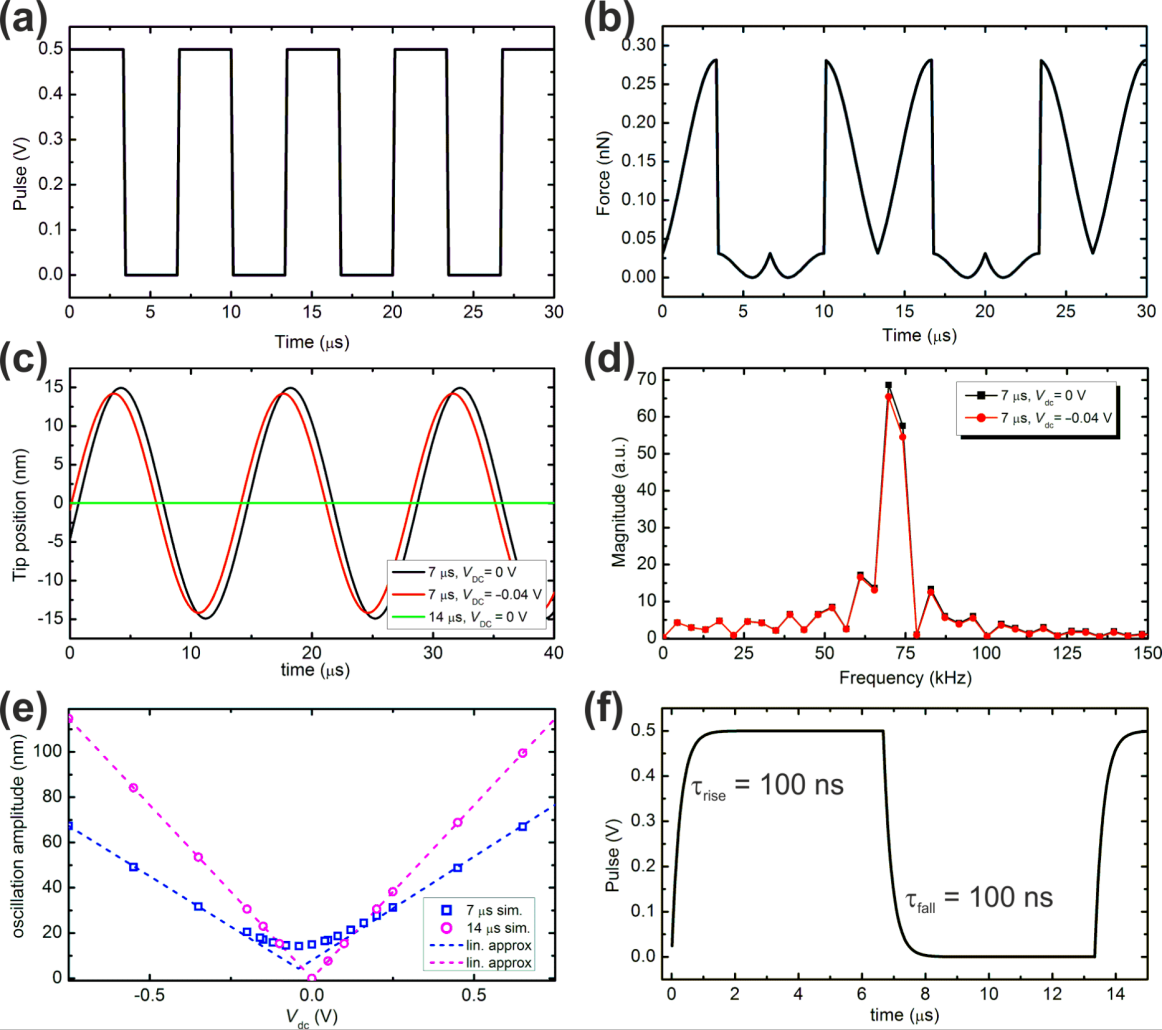
(a) Perturbation signal with a square pulse shape, exemplarily shown for a period of 6.67 μs, a pulse width of ½ the period (3.33 μs), and an amplitude of 0.5 V. (b) Total electrostatic driving force acting on the cantilever for the application of the above pulse plus a dc-bias voltage of −250 mV. (c) Tip oscillation for three different conditions as indicated in the legend, showing that not in all cases a dc voltage can be found to reduce the tip oscillation to zero. (d) FFT spectra of the tip oscillation for the two cases shown in (c). (e) Simulated oscillation amplitude as a function of applied dc voltage showing the expected v-shape of the amplitude. (f) Pulse shape with exponential raise (τ_rise_ = 100 ns) and fall (τ_fall_ = 100 ns) used in some of the presented simulations.

Figure 1a shows a typical bias modulation applied to the sample in the simulations with a pulse period of 6.67 μs, corresponding to twice the cantilever’s fundamental frequency, with a duration of 50% of the period. Considering this modulation voltage and the addition of a dc-bias voltage of −250 mV (added in order to offset the time average of the bias modulation), the total time-dependent driving force acting on the cantilever according to Equations 2–4 is shown in Figure 1b. The shape of this driving force is rather complex. The tip–cantilever motion is affected by this driving force, leading to an imperfect compensation of the electrostatically excited oscillation in some cases. Figure 1c shows the tip oscillation for three different cases. For pulses applied at the same frequency as the ac bias, the oscillation amplitude can be effectively reduced to zero for the expected CPD. However, for the case where the pulse frequency is twice the resonance frequency, this condition cannot be achieved by any dc voltage. The tip–cantilever motion can be analyzed in the form of a fast-Fourier transformation (FFT), namely FFT[*f*_dc_(*t*) + *f*_ac_(*t*) + *f*_2ac_(*t*)], as shown in Figure 1d. Due to the complexity in the shape of the driving forces, the FFT may be non-zero at the detection frequency even if the applied dc voltage equals the time average of the pulse, as in these particular cases. Specifically, one would expect that the application of a dc bias of −250 mV should nullify the *f*_ac_ component of the driving force *F*_ac_ of Equation 3, if the modulation frequency is much faster than the Kelvin controller. However, this is only true if the FFT of the total driving force is zero at *f*_ac_, which Figure 1d shows is not the case for this example. The complexity of the driving forces, whereby the usual KPFM driving forces (*F*_dc_, *F*_ac_, and *F*_2ac_) are convoluted with the voltage pulse, makes it difficult to draw analytical generalizations beyond the observation of the value of the FFT at the detection frequency. Figure 1e shows the obtained tip oscillation amplitudes as a function of the applied dc voltage for the cases illustrated in Figure 1c. When the pulse frequency equals *f*_ac_, the oscillation amplitude reduced to zero for the expected CPD. However, at pulse frequency twice the ac frequency, the amplitude cannot be reduced to zero and exhibits a minimum at a dc voltage different from the expected CPD.

**Figure 2:**
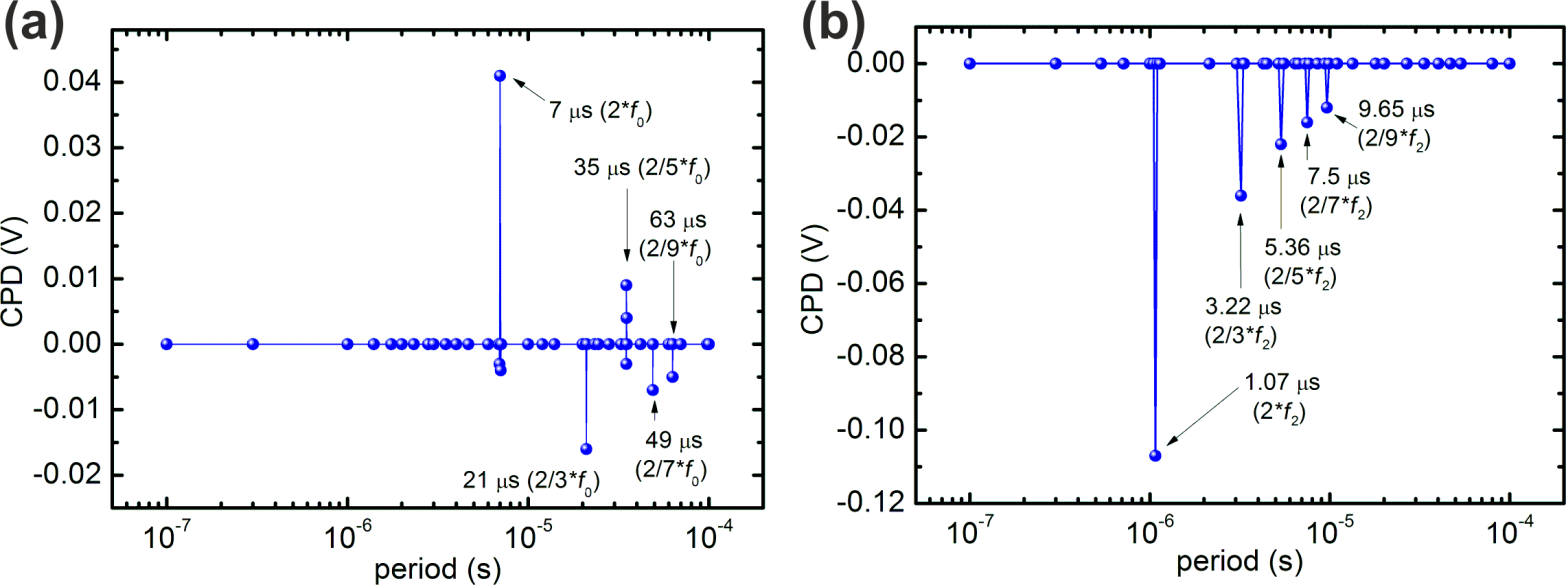
Frequency spectra for square pulse perturbation signal (*V*_pulse_ = 0.5 V, w_pulse_ = ½ period) with the detection ac frequency on the (a) fundamental cantilever eigenmode (*f*_ac_ = *f*_0_ = 71.429 kHz) and (b) second cantilever eigenmode (*f*_0_ = 74.626 kHz, *f*_ac_ = *f*_2_ = 466.4125 kHz). In both cases *V*_ac_ = 0.5 V was used, along with a dc bias of −250 mV.

In typical experiments of time-resolved KPFM [22,24–25,27–28,30], spectra as a function of the modulation frequency are recorded. We simulated the spectral dependence of the CPD as a function of the period of the modulated bias. In our simulations, the amplitude of the modulated bias is set to 0.5 V and the period is changed over a range of 3 orders of magnitude from 100 ns to 100 μs. This range was chosen as it contains the frequencies of the fundamental and second resonance mode, as well as many of their multiples. Figure 2 shows the result of simulations where the preset dc bias was set to −0.25 V, such that the time averaged square bias pulse leads to an expected measured CPD of 0 V. Over most of the spectral range, the expected CPD is indeed obtained. However, in the case of using the fundamental resonance *f*_0_ several deviations are observed (see Figure 2a). A strong deviation is seen at a modulation period of 7 μs, corresponding to twice the selected fundamental resonance frequency of 71.429 kHz, and therefore equal to twice the Kelvin detection ac-frequency. Smaller deviations are observed at the frequencies 2/m·*f*_0_, where *m* is an odd number. The strong deviation at 2·*f*_0_ can be attributed to a capacitive coupling according to Equation 4. Analysis of the total electrostatic driving force for the cases where the pulse frequency is 2/*m*·*f*_0_ (not shown) reveals that the shape of the driving force contains similar features in all cases, and the FFT analysis confirms that there is indeed a non-zero driving force at the detection frequency in all cases, with its magnitude decreasing as *m* increases.

Experimentally, the frequency of the second resonance mode of the cantilever is often used to apply the Kelvin ac-detection voltage: *f*_ac_ = *f*_2_ [2]. We therefore also simulated the corresponding case, which is shown in Figure 2b. As in the above case, a deviation from the expected CPD is observed at 2/*m*·*f*_2_, while no additional deviations are detectable. We note that there is also no deviation detected at the fundamental resonance frequency, indicating that the pure capacitive excitation of the cantilever at its fundamental resonance does not have any impact on the CPD measurement.

**Figure 3:**
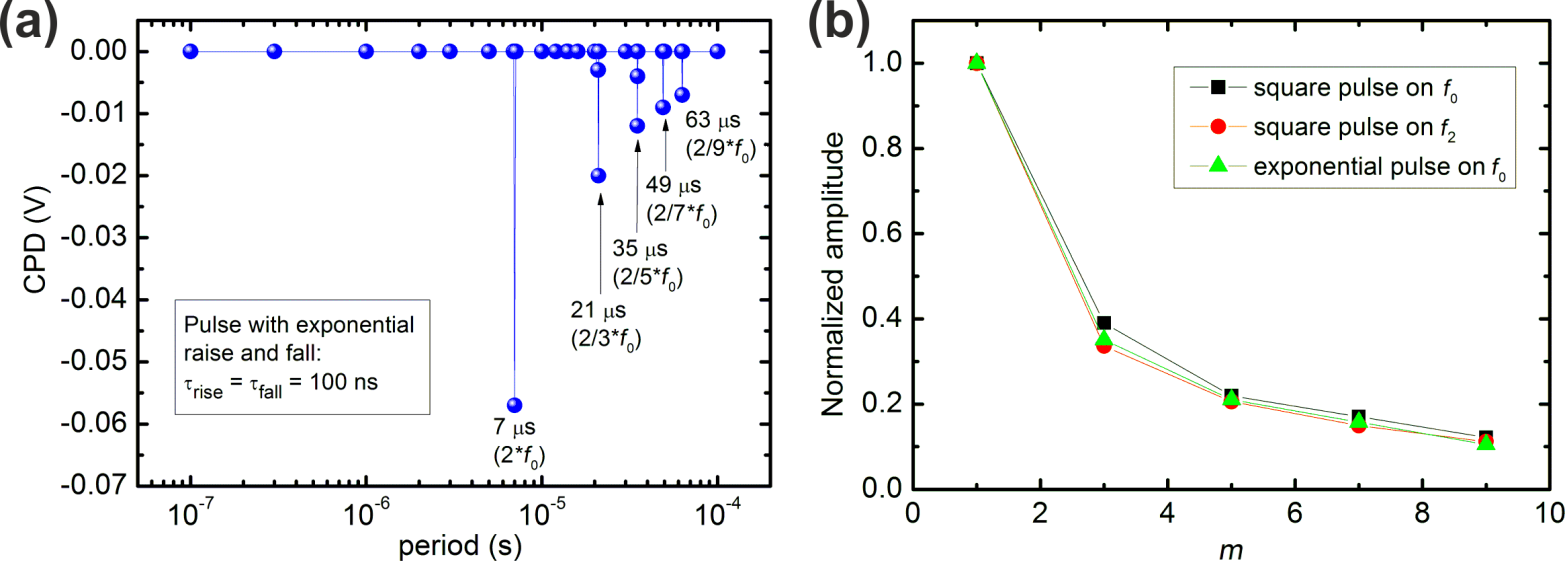
(a) Frequency spectrum for bias modulation with exponential rise and fall shape, as illustrated in Figure 1f. The characteristic time constant of the exponential rising and falling slopes are τ_rise_ = τ_fall_ = 100 ns. The pulse height and width are *V*_pulse_ = 0.5 V and w_pulse_ = ½ period, respectively. As in previous cases, a dc-bias voltage of −250 mV is included. The detection ac frequency was applied at the fundamental cantilever resonance (*f*_ac_ = *f*_0_ = 71.429 kHz). (b) Normalized amplitude of the CPD deviations as a function of the factor *m* (see text for details).

In addition to a square pulse shape of the modulation bias, we also considered pulses with an exponential rise and fall (see Figure 1f), a case which resembles the effect of charge generation, separation, and recombination. Thus, the pulse shape with exponential rise and fall is closer to what is expected to be observed in an experiment [25,30]. Figure 3a shows the resulting simulated frequency spectrum of a modulated bias with exponential rise and fall slopes with a characteristic time constant of τ_rise_ = τ_fall_ = 100 ns, and a dc bias of −250 mV. The obtained spectrum shows deviations from the expected CPD at the same modulation frequencies as in the case of the square bias pulses, i.e., at *f*_mod_ = 2/*m*·*f*_0_ (with *m* = 1,3,5,7,…). Thus, frequency mixing also affects the simulated CPD for modulated bias pulses with exponential rise and fall shape. The deviation from the expected CPD shows a systematic dependence on the factor *m*, where the deviation is largest when the modulation frequency corresponds to twice the frequency of the ac detection frequency of the Kelvin signal. Figure 3b shows the normalized amplitude of the simulated spectra of Figure 2a,b, and Figure 3a, which are in agreement with FFT analyses.

### Experiments

To corroborate the results from the simulations, we carried out experiments of tr-KPFM with bias and light pulses on a model system consisting of a Au(111) sample. Different experimental conditions were realized to reflect the different simulation scenarios and also typical experimental conditions.

**Figure 4:**
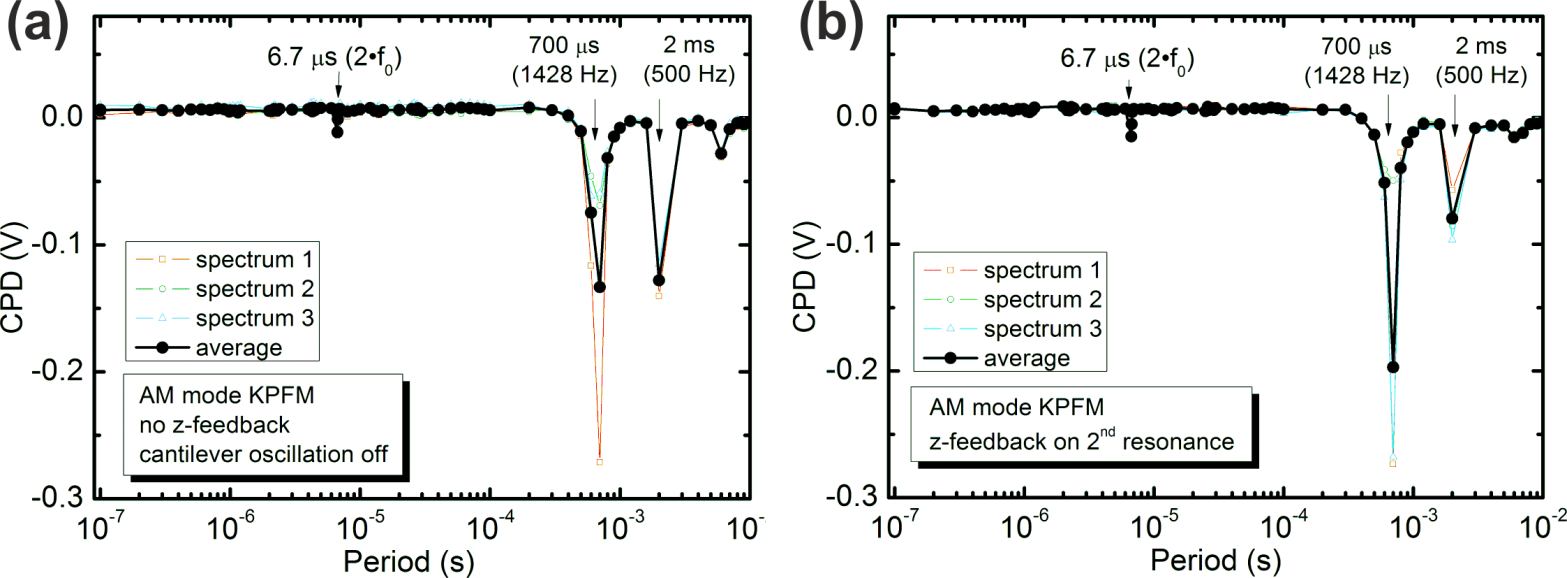
Experimental frequency spectra with a square shaped bias pulse (*V*_pulse_ = 0.2 V) of 50% duty cycle. (a) Spectrum taken with the *z* feedback and the cantilever oscillation switched off and (b) with the cantilever mechanical oscillation at the 2nd resonance mode (*f*_2_ = 465.207 kHz) and *z* feedback switched on. In both cases the Kelvin detection ac bias (*V*_ac_ = 0.2 V) is applied at the fundamental resonance frequency (*f*_0_ = 73.680 kHz).

In the above numerical simulations, the tip–sample distance is maintained constant (the *z* feedback is inactive) and the ac-bias voltage is applied at the fundamental resonance frequency of the cantilever (results presented in Figure 2), while a frequency spectrum with a square pulse shape is applied. Experimental results reproducing these conditions are presented in Figure 4a; AM-KPFM was used at the fundamental resonance frequency of the cantilever. The detailed experimental conditions are given in the figure caption. During the acquisition of the frequency spectrum, the *z* feedback was deactivated and the cantilever oscillation was turned off. Thus, any cantilever oscillation is only induced by the ac-bias voltage when acquiring the frequency spectrum. Figure 4a shows the average (black curve) of 3 individual spectra (colored thin curves) taken under the same conditions at the same sample location. The expected relative CPD in the experiment was *V*_CPD_ = 0 V and a clear deviation is observed at 1/*f*_ac_ = 6.7 μs, corresponding to twice the resonance frequency of the cantilever (*f*_0_ = 73.680 kHz). This finding is in agreement with the results from the simulation (see Figure 2a). However, the simulations show an additional deviation (about 4–5 times smaller) at 2/3 times the resonance frequency of the cantilever, where the experimental data does not show this deviation from the expected CPD. Considering the small deviation observed at 2·*f*_0_, it is possible that the deviation at 2/3·*f*_0_ may be within the noise level of the experimental data. Nevertheless, two additional deviations from the expected CPD are found at frequencies not related to the cantilever eigenmodes, namely at 500 Hz and at 1428 Hz. We speculate that these deviations are due to possible capacitive cross talk between the ac voltage and the piezo cables or the photodetector. We can disregard these deviations since they are not related to the cantilever eigenmodes and have a different origin, possibly related to the specific setup used for the experiments.

As the above experimental conditions are far from a realistic experiment, we also performed experiments with an active cantilever oscillation and active *z* feedback. Since the fundamental resonance is used for the ac bias of the Kelvin setup, we chose to use the second eigenmode of the cantilever for the mechanical oscillation and the *z* feedback. The results are shown in Figure 4b and are in excellent agreement with the experiment where the cantilever oscillation and *z* feedback were switched off (Figure 4a). As detailed above, they are also in agreement with the simulation results, thus demonstrating that neglecting the cantilever oscillation and *z* feedback in the simulations does not have a significant impact on the results, which confirms the validity of the simulations.

While the above experiments aim at reproducing the conditions used in the simulations presented in the previous section, a typical experiment would use the fundamental eigenmode for the *z* feedback and either use the second eigenmode for the Kelvin feedback in AM-KPFM (by applying the detection ac bias on the second eigenmode) or use FM-KPFM and apply an ac bias for the Kelvin detection in the range below ≈1 kHz. We thus used FM-KPFM to test the effect of the frequency spectra for time-resolved KPFM in realistic experimental conditions.

**Figure 5:**
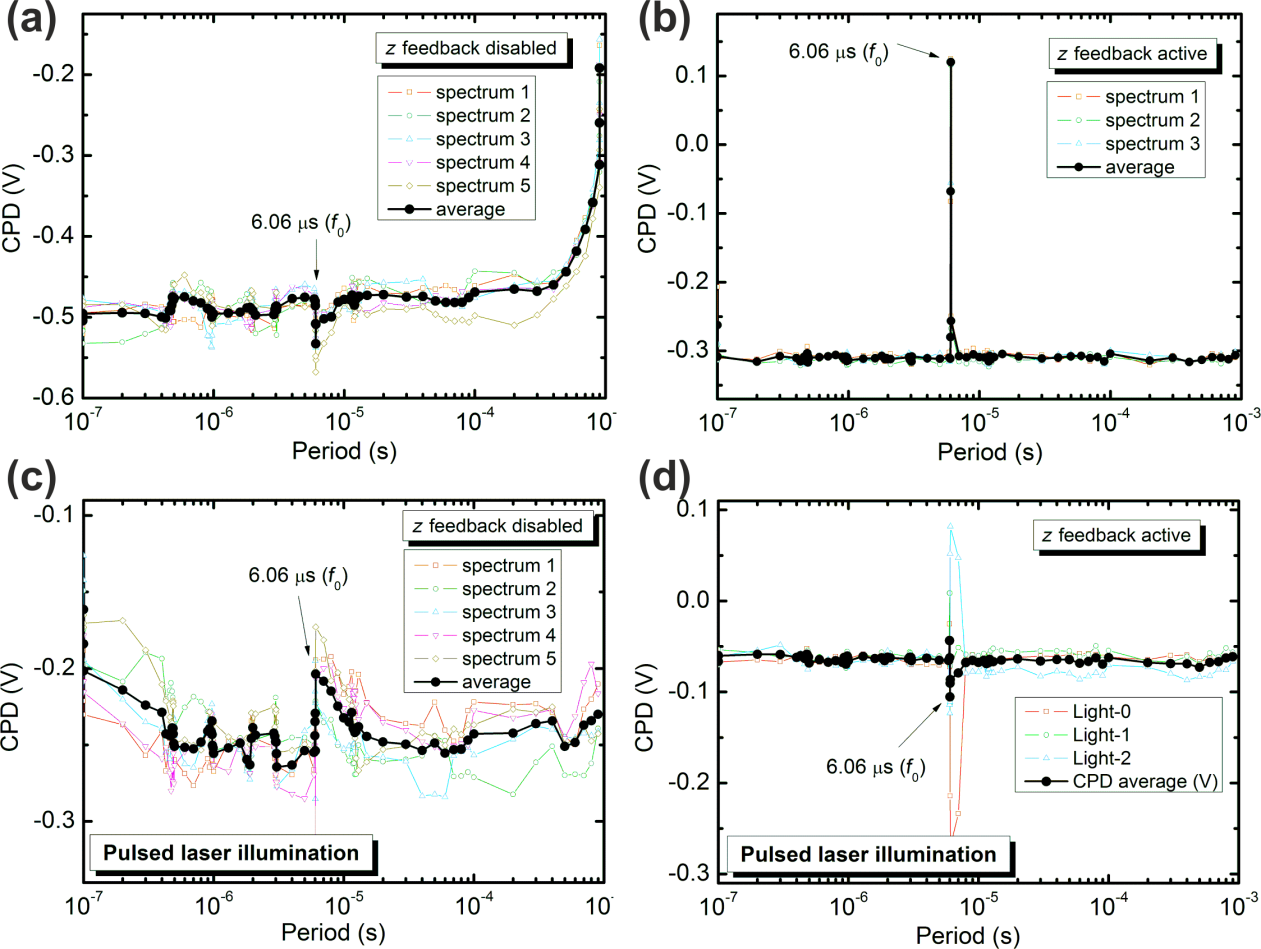
Experimental frequency spectra with a square shaped pulse of 50% duty cycle measured by FM-KPFM (*f*_ac_ = 377 Hz, *V*_ac_ = 200 mV). Bias pulses applied to the Au(111) sample with the *z* feedback (a) disabled and (b) active, leading in both cases to some deviation from the expected CPD at the fundamental resonance frequency of the cantilever. Light pulses lead to similar deviations for (c) disabled and (d) enabled *z* feedback.

For the experiments, a cantilever with a fundamental resonance frequency of *f*_0_ = 165.448 kHz was used and the ac-bias voltage for the FM-KPFM detection was applied at 377 Hz with *V*_ac_ = 200 mV. A frequency spectrum with a square-shaped bias pulse was applied with 50% duty cycle. In case of a disabled *z* feedback, the spectrum shows a slight deviation from the otherwise nearly featureless CPD spectrum at the fundamental resonance frequency, as shown in Figure 5a. In the case of an active *z* feedback, the deviation from the expected CPD at the fundamental resonance frequency is significantly larger, as shown in Figure 5b. This much stronger deviation is due to a strong impact on the cantilever oscillation, which leads to a sizable effect on the *z* feedback and thus the tip–sample distance, as was confirmed by monitoring the *z*-piezo position. Therefore, the origin of the artifact in the frequency spectrum in the present case is different from the case shown in Figure 4, where no effect on the *z*-piezo position was observed.

Several research results on time-resolved KPFM have been presented that used intensity-modulated (IM) laser illumination to trigger a SPV change and thereby study the dynamics of charge generation, separation, and recombination. To investigate potential artifacts of IM laser illumination we also carried out similar experiments to the above frequency spectra with a IM laser instead of applying a pulsed bias. As the experiments use an Au(111) sample and a PtIr-coated tip and cantilever, no SPV due to the laser illumination is expected. However, as seen in Figure 5c and Figure 5d, a clear deviation from the flat CPD spectrum is again observed at the fundamental resonance frequency of the cantilever. This artifact can be explained by considering that part of the IM laser illumination reflects from the cantilever back side and the sample onto the position sensitive photodetector for the AFM detection, thereby leading to an artificial modification of the measured cantilever oscillation. We could confirm an impact of the IM laser on the tip–sample distance by monitoring the *z*-piezo position, which presumably leads to the detected deviation from the expected CPD. It is noteworthy that in the case of IM laser light and an active *z* feedback we observed some variation in the size and sign of the deviation from the expected CPD value (see, e.g., thin colored lines in Figure 5). We attribute this variation to a different phase shift between the cantilever oscillation and the IM laser, leading to constructive or destructive interference between the AFM detection laser and the IM illumination. We note that a shift of the measured CPD with a variation of the phase between a mechanical and an electrostatic modulation signal at the fundamental resonance frequency has been reported previously in conjunction with the so-called dissipation KPFM mode [31–32].

It is also important to note that the artifacts in time-resolved KPFM observed in Figure 4 and Figure 5 have a different origin from one another. In the latter case, a direct interference of the applied bias or light pulses with the cantilever oscillation leads to deviations in the measured oscillation and (in case of an active *z* feedback) the tip–sample distance, thereby influencing the CPD controller. In the experiments presented in Figure 4, the observed artifact results from a frequency mixing of the applied bias pulses with the KPFM ac detection voltage, and not directly from an influence on the cantilever oscillation at the resonance frequency. In any experiment, both artifacts must be considered, and can be avoided by excluding the resonance frequency which is used for the *z* feedback and frequencies related to the KPFM detection frequency. It is also a good practice to perform the FFT of the total electrostatic driving force corresponding to Equations 2–4 for the particular pulse configurations used.

## Conclusion

We have performed an analysis of possible artifacts in time-resolved KPFM using a combined simulation and experimental study. The simulations of time-resolved CPD spectra revealed a deviation from the expected CPD at fractions of twice the ac detection frequency used for the CPD. The experiments confirmed these observations. Furthermore, in typical experimental measurement conditions, additional artificial CPD deviations can appear due to a direct cross talk of the modulation signal (bias, light, etc.) with the cantilever oscillation. Our results provide the following guidelines to avoid artifacts in time-resolved KPFM measurements: (i) during the acquisition of CPD spectra, application of the modulation signal at the critical frequencies should be avoided, and (ii) an FFT analysis of the total electrostatic driving force is recommended to avoid misinterpretation of experimental data.

## Experimental

Experiments were performed in an ultra-high vacuum atomic force microscope (Omicron VT-SPM) at a base pressure below 1 × 10^−10^ mbar, controlled by a Nanonis controller. Two types of PtIr-coated cantilevers (Nanosensors PPP) were used, with the fundamental resonance frequency at ≈74 kHz or at ≈165 kHz. Typical oscillation amplitudes of ≈20 nm were mechanically excited. For amplitude modulation (AM) KPFM the second resonance mode of the cantilever was used (*f*_2_ ≈ 465 kHz or *f*_2_ ≈ 1.027 MHz, respectively), while for frequency modulation (FM) KPFM the ac bias was applied at *f*_ac_ = 377 Hz. In both cases *V*_ac_ = 200 mV amplitude was used. The induced oscillating electrostatic forces are compensated by a dc voltage that corresponds to the contact potential difference (CPD).

The topography control (*z* feedback) was normally realized on the fundamental resonance of the cantilever. However, in some experiments the *z* feedback and the cantilever oscillation were switched off during the pulse sequences and the tip was retracted 50 nm. Control experiments with different tip retractions between 10 and 50 nm showed no dependence of the results on the tip retraction. To allow Kelvin control on the fundamental resonance frequency (by applying the Kelvin ac voltage at *f*_0_) the topography control was applied on the second resonance mode of the cantilever. The specific experimental conditions are stated in the results section.

Time-resolved KPFM was realized by applying bias pulses from a pulse generator (Agilent MSO-X-3014A) to a metallic Au(111) sample with periods ranging from 100 ns to 1 ms and a duty cycle of 50%. Thus the shortest pulses realized in the present experiments were 50 ns. Using a metallic Au sample ensures that carrier dynamics are not relevant in the present study.
